# RETRACTION: Exploring the amino acid profile and microbial properties of locally sweet preserved kachra hareer (*Terminalia chebula*)

**DOI:** 10.1002/fsn3.4172

**Published:** 2024-05-02

## Abstract

**RETRACTION:** Ali Ikram, Farhan Saeed, Haroon Munir, Muhammad Tauseef Sultan, Muhammad Afzaal, Aftab Ahmed, and Faqir Muhammad Anjum, “Exploring the amino acid profile and microbial properties of locally sweet preserved kachra hareer *(Terminalia chebula)*.” *Food Science & Nutrition*, *9* (2021): 909–919. https://doi.org/10.1002/fsn3.2056.

The above article, published online on 13 December 2020 in Wiley Online Library (wileyonlinelibrary.com), has been retracted by agreement between the journal's Editor‐in‐Chief, Y. Martin Lo, and Wiley Periodicals, LLC. The retraction has been agreed to following concerns about the peer review process. The editorial office found unambiguous evidence that the manuscript was accepted solely based on compromised and insufficient reviewer reports. This evidence was further investigated and verified by the publisher and the Editor‐in‐Chief. As a result, the conclusions of this article are not considered reliable. The authors were notified of the retraction and disagreed with this decision.

**RETRACTION:** Ali Ikram, Farhan Saeed, Haroon Munir, Muhammad Tauseef Sultan, Muhammad Afzaal, Aftab Ahmed, and Faqir Muhammad Anjum, “Exploring the amino acid profile and microbial properties of locally sweet preserved kachra hareer *(Terminalia chebula)*.” *Food Science & Nutrition*, *9* (2021): 909–919. https://doi.org/10.1002/fsn3.2056.

The above article, published online on 13 December 2020 in Wiley Online Library (wileyonlinelibrary.com), has been retracted by agreement between the journal's Editor‐in‐Chief, Y. Martin Lo, and Wiley Periodicals, LLC. The retraction has been agreed to following concerns about the peer review process. The editorial office found unambiguous evidence that the manuscript was accepted solely based on compromised and insufficient reviewer reports. This evidence was further investigated and verified by the publisher and the Editor‐in‐Chief. As a result, the conclusions of this article are not considered reliable. The authors were notified of the retraction and disagreed with this decision.

